# Analgesic benefits of pre-operative versus postoperative transversus abdominis plane block for laparoscopic cholecystectomy: a frequentist network meta-analysis of randomized controlled trials

**DOI:** 10.1186/s12871-023-02369-6

**Published:** 2023-12-12

**Authors:** Burhan Dost, Alessandro De Cassai, Eleonora Balzani, Federico Geraldini, Serkan Tulgar, Ali Ahiskalioglu, Yunus Emre Karapinar, Müzeyyen Beldagli, Paolo Navalesi, Cengiz Kaya

**Affiliations:** 1 Department of Anesthesiology and Reanimation, School of Medicine, Ondokuz Mayis University Faculty of Medicine, Kurupelit, Samsun, TR55139 Turkey; 2 UOC Anesthesia and Intensive Care Unit “Sant’Antonio”, University Hospital of Padua, Padua, Italy; 3 Department of Surgical Science, University of Turin, Torino, Italy; 4 Department of Anesthesiology and Reanimation, Samsun Training and Research Hospital, Samsun University Faculty of Medicine, Samsun, Turkey; 5 Department of Anesthesiology and Reanimation, Ataturk University School of Medicine, Erzurum, Turkey; 6 Clinical Research, Development and Design Application and Research Center, Ataturk University School of Medicine, Erzurum, Turkey; 7 UOC Institute of Anesthesia and Intensive Care Unit, University Hospital of Padua, Padua, Italy; 8 DIMED Department of Medicine, University of Padua, Padua, Italy

**Keywords:** Laparoscopic cholecystectomy, Nerve block, Meta-analysis, Anesthesia, analgesia, Analgesics, Ultrasonography

## Abstract

**Background:**

Transversus abdominis plane (TAP) block has been utilized to alleviate pain following laparoscopic cholecystectomy (LC). However, the optimal timing of administration remains uncertain. This study aimed to compare the efficacy of pre-operative and postoperative TAP blocks as analgesic options after LC.

**Methods:**

A frequentist network meta-analysis of randomized controlled trials (RCTs) was conducted. We systematically searched PubMed (via the National Library of Medicine), EMBASE, Scopus, Cochrane Central Register of Controlled Trials (CENTRAL), and Web of Science up to March 2023. The study included RCTs that enrolled adult patients (≥ 18 years) who underwent LC and received either pre-operative or postoperative TAP blocks. The primary outcome assessed was 24-hour postoperative morphine consumption (mg). Additionally, pain rest scores within 3 hours, 12 hours, and 24 hours, as well as postoperative nausea and vomiting (PONV), were considered as pre-specified secondary outcomes.

**Results:**

A total of 34 trials with 2317 patients were included in the analysis. Postoperative TAP block demonstrated superiority over the pre-operative TAP block in reducing opioid consumption (MD 2.02, 95% CI 0.87 to 3.18, I2 98.6%, *p* < 0.001). However, with regards to postoperative pain, neither pre-operative nor postoperative TAP blocks exhibited superiority over each other at any of the assessed time points. The postoperative TAP block consistently ranked as the best intervention using SUCRA analysis. Moreover, the postoperative TAP block led to the most significant reduction in PONV.

**Conclusions:**

The findings suggest that the postoperative TAP block may be slightly more effective in reducing 24-hour postoperative opioid consumption and PONV when compared to the pre-operative TAP block.

**Trial registration:**

PROSPERO, CRD42023396880.

**Supplementary Information:**

The online version contains supplementary material available at 10.1186/s12871-023-02369-6.

## Introduction

Laparoscopic cholecystectomy (LC) is considered a minimally invasive surgical procedure compared to traditional open cholecystectomy [1, 2]. However, although less severe in LC, procedure-related pain remains a problem that needs to be addressed by clinicians [1, 3]. Pharmacological treatments, such as nonsteroidal anti-inflammatory drugs and opioids, are frequently used in pain management in LC [4, 5]. Also, loco-regional anesthesia techniques, such as port site infiltration and fascial plane blocks, can improve the quality of pain management [3]. The transversus abdominis plane (TAP) block is one of the first fascial plane blocks for pain management after various surgical procedures, including laparoscopic surgeries.

The TAP block aims to provide sensory blockage of the anterior abdominal wall and generally does not affect visceral pain [6, 7]. Numerous studies have been published on the peri-operative analgesic efficacy of TAP block types (because there is more than one approach) in LC [8–10]. In many studies on regional anesthesia, the aim was to measure the effectiveness of the blocks. In these studies, measurements are usually set up as parameters such as opioid requirement, pain scores, and healing quality scores at a specific time interval [7]. However, the parameters affecting the results of a study are not limited to block characteristics, such as block type, local anaesthetic volume, and concentration. Comparisons are generally made between the control and experimental groups or between more than one experimental group.

Another critical factor is whether the applied regional anesthesia technique is performed before or after the surgical procedure, i.e., whether it provides pre-emptive/preventive analgesia. The common goal in pre-emptive analgesia applications such as pre-operative TAP block is to reduce the intensity and duration of postoperative pain by preventing central sensitization and reducing peripheral nociceptive input [11]. Based on the data revealing that the application of regional anesthesia to be performed early reduces the immune response to perioperative trauma, debates about whether to apply a pre- or postsurgical block are still ongoing [12, 13].

Therefore, we conducted a meta-analysis of previously published randomised controlled trials to compare the effects of pre-operative and postoperative TAP blocks in LC. Our hypothesis was that a pre-operative TAP block would reduce morphine consumption by providing pre-emptive analgesia and allowing more time for LA to diffuse in the fascial plane.

## Methods

### Eligibility criteria, literature search, and study selection

This systematic review and meta-analysis followed the steps outlined by the Preferred Reporting Items for Systematic Reviews and Meta-Analyses (PRISMA) statement [14]. We followed the PRISMA extension statement for reporting systematic reviews incorporating network meta-analyses of health care interventions: checklist and explanations [15]. The Cochrane Handbook for Systematic Reviews of Intervention was chosen as the methodological guidance [16]. The protocol was registered prospectively in PROSPERO (CRD42023396880). We defined inclusion criteria using the PICOS acronym items: adult patients (≥ 18 years) who underwent LC (P) who received pre-operative or postoperative ultrasound guided TAP blocks including all approaches reported in the literature for TAP block [17] (I) compared to placebo or no intervention (C). Our primary outcome was 24-hour postoperative morphine consumption (mg). For all papers expressing the cumulative postoperative opioid dose with a drug other than morphine, we converted the amount following the equi-analgesic tables using the GlobalRPh morphine equivalent calculator, considering a 0% cross-tolerance modifier (http://www.globalrph.com/narcotic). Our pre-specified secondary outcomes were pain rest scores within 3 hours and at 12 and 24 hours expressed through the visual analogue scale (VAS) or numerical rating scale (NRS), and postoperative nausea and vomiting (PONV), defined as a self-reported outcome in the first 24 postoperative hours (O) [18]. We decided to include only RCTs (S). Regarding the TAP block timing we defined pre-operative block as a TAP block performed before the surgical incision. All blocks performed after surgical incisions were defined as postoperative blocks. We did not exclude studies based on the type of local anaesthetic and volume injected.

We performed an electronic search of PubMed (via the National Library of Medicine), EMBASE, Scopus, Cochrane Central Register of Controlled Trials (CENTRAL), and Web of Science from database inception to 8 March 2023 using a predefined search strategy (Supplementary Document 1). The research strategy was decided and approved in advance by regional anesthesia experts. Language restrictions were not imposed. In this review, two reviewers (Y.E.K. and M.B.) independently identified and assessed studies to determine if they were eligible, and a third reviewer settled any disagreements that arose (B.D.). Two authors extracted data and evaluated the potential for bias separately (F.G. and A.D.C.).

### Data extraction and risk-of-Bias assessment

Data for this systematic review were extracted using an Excel (Microsoft, Redmond, WA, USA) spreadsheet that had been specifically prepared for this purpose. Data regarding the procedure, including the year of publication, the patient’s age, the patient’s American Society of Anaesthesiologists (ASA) physical status score, the type local anaesthetic used, the injection volume, the amount of local anaesthetic used, and the TAP block technique, used was collected. Two researchers used the Risk of Bias (RoB) 2 instrument to assess the reliability of the included RCTs [16]. Randomization, allocation concealment, blinding of outcome assessment, data completeness, and selective outcome reporting were used to assign each study a risk of bias grade of either low, high, or with some concerns. The certainty and quality of the evidence for each outcome was assessed using the Grading of Recommendations Assessment, Development, and Evaluation (GRADE) methodology [19]. To check for publication bias, we generated funnel plots, and we intended to conduct additional statistical analysis of funnel plot asymmetry if more than 10 trials were evaluated for each outcome [16]. Evaluations of homogeneity, consistency, and intransitivity are given in the section on statistics that follows.

### Statistical analysis

Data meta-analysis was conducted using the R package “netmeta” in R version 4.1 (R Foundation for Statistical Computing, Vienna, Austria). We used the χ2 test and the I2-statistic (classifying I2 values as low (25%), moderate (25–50%), or high (> 50%) to evaluate the degree of heterogeneity within the studies [19]. We used mean differences (MDs) with 95% confidence intervals (CIs) to describe the treatment’s impact on continuous variables. For binary outcomes, we report the effect as an odds ratio (OR) with 95% CI.

We ranked all treatments in terms of their ability to reduce 24-hour morphine consumption, from 0 to 1, using the surface under cumulative ranking (SUCRA) curve [20]. A higher SUCRA (values closer to 1) suggests that a given treatment is more likely to be optimal for the outcome of interest. We were able to rank the treatments by comparing the SUCRA values that were calculated for each. Whenever possible, we used the Hozo method to transform the reported median and interquartile range into an estimated mean and standard deviation (SD) [21]. When different doses of local anaesthetic were used in the same study for the same block, the means and standard deviations were pooled.

Cochrane’s Q test was used to assess the degree of heterogeneity within and between studies. Despite the inconsistency and heterogeneity, a random-effects model was chosen. We inspected funnel plots visually and used the Egger test (p 0.05, indicating possible publication bias) to assess publication bias (Supplementary Document 2). All statistical significance testing was 2-tailed; a *p* value of < 0.05 was considered statistically significant.

## Results

**Fig. 1:**
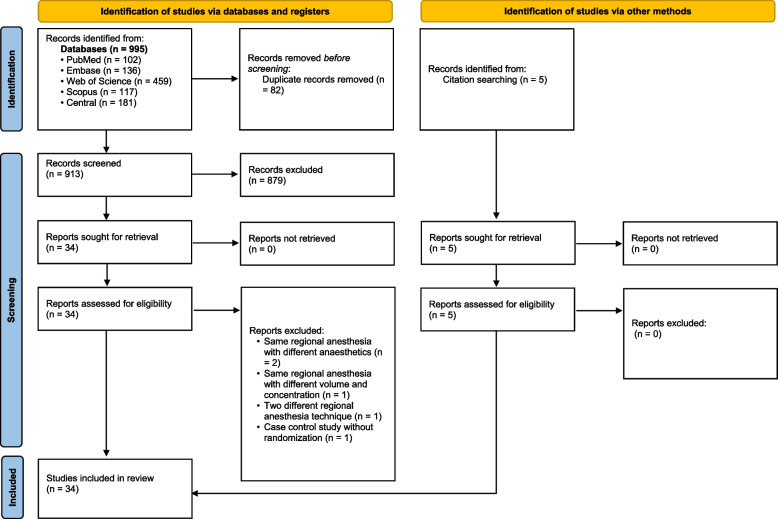
PRISMA flow diagram. The diagram illustrates the study selection process and provides reasons for excluding records during the screening. PRISMA, Preferred Reporting Items for Systematic Reviews and Meta-Analyses

The search results are presented in the PRISMA flow diagram (Fig. 1). Initial screening identified 995 studies. Of these, 82 search results were excluded during the preliminary screening because they were duplicates, and 879 were excluded because they were unrelated to the topic. The full texts were retrieved from the remaining 34 articles, and five more studies were excluded according to our inclusion and exclusion criteria. Five additional studies were identified by screening the references of the included articles. A total of 34 studies were included in the quantitative analysis [11, 22–54]. Table 1 summarises the pooled characteristics of the included studies.

**Table 1:** Summary of findings table

Author (Year)	Country	Age	ASA	LA1	LA2	Block Timing	GA protocol	PO analgesia protocol	Main Outcome
Rahimzadeh P (2022) [11]	Iran	20–60	I-II	20 ml of ropivacaine 0.25%	20 ml of ropivacaine 0.25%	Preoperative vs. Postoperative	PREMEDICATION: FNT 2 μg/kg, MDZ 0.12 mg/kg INDUCTION: PRP 2 mg/ kg and cisatracurium 0.2 mg/kg MAINTENANCE: Isoflurane 1MAC (1.2%), cisatracurium 2 mg every 30 min	PCA: 20 mg/ml of Acetaminophen and 0.6 mg/ml of Ketorolac with a bolus of 2 ml every 15 min	Postoperative Pain
Arik E (2020) [22]	Turkey	18–80	I-III	20 mL 0.25% bupivacaine	–	Preoperative	PREMEDICATION: MDZ 0.03 mg/kg INDUCTION: PRP 2.5 mg/kg; FNT 1 mcg/kg; ROC 0.6 mg/kg MAINTENANCE: SEVO 1% MAC	PCA: tramadol (5 mg/mL) 20 mg bolus, 20 min lock-out time, and a maximum infusion rate of 200 mg in 4 h without basal infusion. Dexketoprofen 50 mg IV for rescue when NRS > 4	Postoperative Pain
Dost B (2018) [23]	Turkey	20–70	I-II	levobupivacaine 0.5% 15 mL+ saline 15 mL	–	Preoperative	PREMEDICATION: MDZ 0.03 mg/kg INDUCTION: PRP 2.5 mg/kg; FNT 1 mcg/kg; ROC 0.6 mg/kg MAINTENANCE: SEVO 1% MAC	PCA: tramadol (5 mg/mL) 20 mg bolus, 15 min lock-out time, and a maximum infusion rate of 200 mg in 4 h with basal infusion of 5 mg/h. Meperidin 0.5 mg/kg IV for rescue when NRS > 4	Postoperative Pain
Bhatia N (2014) [24]	India	18–60	I-II	ropivacaine 0.375% 15 ml	–	Postoperative	PREMEDICATION: alprazolam 0.25 mg + ranitidine 250 mg; INDUCTION: morphine 0.1 mg/kg PRP 2–3 mg/kg vecuronium 0.1 mg/kg; MAINTENANCE: N2O 66% +/− Isoflurane 1–2%	IV paracetamol 1 g every 6 hours, VAS score greater than 4, or those requesting analgesic, were given IV tramadol 2 mg/kg; subsequent doses of tramadol, if required, were 1 mg/kg	Postoperative Pain
Ortiz J (2012) [25]	USA	18–64	I-III	ropivacaine 0.5% 15 ml	ropivacaine 0.5% 20 ml	Preoperative	PREMEDICATION: MDZ 1–2 mg, INDUCTION: FNT 2 mcg/kg and PRP 2.5 mg/kg, succinylcholine 1–2 mg/kg or ROC 0.6–1 mg/kg, MAINTENANCE: SEVO, additional boluses of FNT 50 mcg	Hydrocodone 5 mg, acetaminophen 500 mg tablets, 2 tablets for mild pain (NRS 3–5), and morphine 4 mg iv for severe pain (NRS 6–10)	Postoperative Pain
Suseela I (2018) [26]	India	20–65	I-II	bupivacaine 0.25% 20 ml	bupivacaine 0.5% 20 mL (each port 5 mL)	Postoperative	PREMEDICATION: ranitidine 150 mg; metoclopramide 10 mg; MDZ 0.5 mg INDUCTION: PRP 2 mg/kg FNT 2 mcg/kg; succinylcholine 1.5 mg/kg MAINTENANCE: nitrous oxide 66%; isoflurane 0.4 to 0.8%; atracurium 0.5 mg/kg	Paracetamol 1 g at the beginning and 1 g/8 h (postoperative);tramadol 1 mg /kg and diclofenac 1 mg/kg	Postoperative Pain
Vrsajkov V (2018) [27]	Serbia	18–75	I-III	20 mL of 0.33% bupivacaine	–	Preoperative	INDUCTION: PRP (2.5 mg/kg), FNT (3 mcg/kg) and ROC (0.6–0.8 mg/kg) MAINTENANCE: SEVO.	acetaminophen (1 g IV) and morphine (0.1 mg.kg − 1 s), acetaminophen (1 g/8 h IV) and dipyrone (2.5 g/12 h; tramadol (1 mg.kg − 1 /6 h) NPS ≥ 6	Postoperative Pain
Ribeiro KNSA (2019) [28]	India	18–55	I-II	40 mL of 0.35% ropivacaine	–	Preoperative	PREMEDICATION: FNT 2 mcg/kg I.V. INDUCTION: PRP 2 mg/kg IV, 0.1 mg/kg of vecuronium I.V. MAINTENANCE: Vecuronium 0.02 mg/kg iv, air and oxygen and SEVO 1.5–2.0 dial concentration MAC of 1.	paracetamol 1 g IV at extubation and 8th hourly, Tramadol 1 mg/kg was given IV whenever VAS was ≥4	Postoperative Pain
Ra YS (2010) [29]	Korea	20–65	I-II	0.25% levobupivacaine 30 mL	–	Preoperative	PREMEDICATION: none, INDUCTION: glycopyrolate 0.2 mg and MDZ 0.05 mg/kg and introducing 2% PRP and REM 50 μg/ml, ROC 0.6 mg/kg MAINTENANCE: oxygen and nitrous oxide, PRP at 2 μg/ ml	PACU: NRS > 6 ketorolac 30 mg IV, If the pain was not relieved, FNT 20 μg; in WARD: ketorolac 30 mg was injected into all the patients 3 times during postoperative 24 hours for 8 hour	Postoperative Pain
Dai LM (2022) [30]	China	> 18	I-II		20 mL 0.5% ropivacaine	Postoperative	INDUCTION: MDZ (0.05 mg/kg), SUF (0.03 μg/kg), ETM (0.3 mg/kg), and cisatracurium (0.15 mg/kg), MAINTENANCE: PRP (6 mg/ kg/h) and REM (0.3 μg/kg/min)	100 mL of normal saline mixed with SUF (100 μg) and dezocine (20 mg); basal inf. 2 mL/h, bolus dose was 0.5 mL, and a lockout interval of 15 min	Morphine Consumption
Al-Refaey K (2016) [31]	Egypt	18–40	I-II		20 ml volume 0.25 bupivacaine	Preoperative	INDUCTION: PRP 1–1.5 mg/kg, FNT 1 μ/kg, and atracurium 0.5 mg/kg, MAINTENANCE: SEVO, 0.4 oxygen/air mixture	morphine (0.02 mg/kg) if VAS score ≥ 4	Recruitment Rate. Adherence Rate. Adverse Events Rate
Lee SY (2022) [32]	Korea	20–70	I-II	40 ml of 0.375% ropivacaine	saline	Postoperative	PREMEDICATION: none, INDUCTION: lidocaine (0.5 mg/kg), PRP (2 mg/ kg), REM (0.5 mcg/kg), and ROC (0.6 mg/kg), MAINTENANCE: oxygen (fraction of inspired oxygen, 0.5), SEVO (2–3%), and REM infusion (0.1 mcg/kg/min)	continuously infuse 0.2 mcg/kg of FNT every hour, and the bolus dose was 0.2 mcg/kg with a 15-min lockout time	Postoperative Pulmonary Function And Analgesia
Jung J (2021) [33]	Korea	20–60	I-III	0.25% ropivacaine	saline	Preoperative	INDUCTION: PRP 4–6 μg/mL and REM 2–6 μg/mL, ROC 0.6–1.0 mg/kg, MAINTENANCE: PRP, REM	PACU: NRS score was > 3, the nurse injected 3-mg IV oxycodone; at ward,30 mg IV ketorolac/8 h at POD0; From POD 1, per-os 50 mg tramadol was administered every 8 hours	Quality Of Recovery
Baral B (2018) [34]	Nepal	18–60	I-II	20 ml of 0.25% Bupivacaine	20 ml of 0.25% Bupivacaine	Postoperative	induction: FNT (2 mcg/kg), PRP (2 mg/kg), and vecuronium (0.8 mg/kg), MAINTENANCE: oxygen, isoflurane, intermittent dose of vecuronium	Paracetamol 1 g 6 hourly; VAS ≥4 pethidine 0.5 mg/kg	Postoperative Pain
Basaran B (2015) [35]	Turkey	18–65	I-II	bupivacaine 0.25% 20 ml	–	Preoperative	PREMEDICATION: MDZ 0.03 mg/kg INDUCTION: FNT 1 μg/kg, MDZ 0.15 mg/kg, PRP 1–2 mg/kg, ROC 1 mg/kg. MAINTENANCE: SEVO, ROC infusion (0.01 mg/kg/min) and FNT (2–3 μg/kg/h).	meperidine 0.5 mg/kg; paracetamol 500 mg po every 6 hours; tenoxicam 20 mg; tramadol 50 mg + 50 mg (when needed) (max dose 500 mg/24 h)	Postoperative Pain
El-Dawlatly AA (2009) [36]	Austria	22–77	I-II	15 mL bupivacaine	–	Preoperative	PREMEDICATION: diazepam 10 mg po INDUCTION: PRP 1–1.5 mg/kg; FNT 2 mcg/kg; ROC 0.6 mg/kg MAINTENANCE: SEVO 1 MAC; REM 0.1 mcg/kg/min	PCA: 1.5 mg bolus morphine without basic rate, 15 min locout time	Postoperative Morphine Consumption
Peterson PL (2012) [37]	Denmark	18–75	I-III	20 mL 0.5% ropivacaine	isotonic saline 0,9% 20 mL	Preoperative	PREMEDICATION: Oral lorezepam 2 mg, INDUCTION: SUF 0.2 mcg/kg, PRP 4 mg/kg, ROC 1 mg/kg MAINTENANCE: SEVO	acetaminofen 1000 mg, every 6 hours, oral ibuprofen 400 mg every 6 hours, iv morphine 5 mg request patient(0–2 hours), 2–24 hours ketobemidone oral 2.5 mg patient own decision	Postoperative Pain
Houben AM (2019) [38]	Belgium	18–75	I-II	levobupivacaine 20 mL 0.375% with epinephrine 5 mcg/mL	saline 20 mL 0.9% with epinephrine 5 mcg/mL	Preoperative	INDUCTION: REM 0,4 mL/kg/h (0.6 mg/mL) PRP, MAINTENANCE: PRP, REM 0.4 mL/kg/h SUF 0.2 mcg/kg	paracetamol 2 g, iv morphine 2 mg bolus,oxycodone 5 mg every 4 hours if necessary	Cumulative Morphine Consumption
Liang M (2020) [39]	China	18–70	I-II	ropivacaine 0.75% 20 mL	20 mL saline 0,9%	Preoperative	INDUCTION: PRP 2 mg/kg, SUF 0.1 mcg/kg, ROC 0.6 mg/kg MAINTENANCE: SEVO 1–2%	parecoxib 40 mg, morphine 2.5 mg for PACU patients, tramadol 100 mg PO for those in the ward	Postoperative Pain
Shin HJ (2014) [40]	USA	18–65	I-II	no block	ropivacaine 20 mL 0.375%	Preoperative	INDUCTION: PRP 1.5 mg/kg, FNT1 mcg/kg, ROC 0.6 mg/kg MAINTENANCE: SEVO	ketorolac 30 mg, if NRS pain score exceeded 6 add FNT 25 mcg,if 4–6, ketorolac 30 mg, on the ward nalbuphine 10 mg (if exceeded 6 or or patient requested)	Postoperative Pain
Breazu C (2022) [41]	Romania	> 18	I-II	bupivacaine 0.25% 20 ml	no block	Preoperative	INDUCTION: PRP 2 mg/kg FNT 2mcg/kg atracurium 0.5 mg/kg or ROC 0.6 mg/kg MAINTENANCE: SEVO		Postoperative Pain
Andic KD (2021) [42]	Turkey	18–75	I-III	bupivacaine 0.375% 20 ml	no block	Preoperative	PREMEDICATION:MDZ 0.03 mg/kg INDUCTION: FNT 2 mcg/kg PRP 1–2 mg/kg ROC 0.6 mg/kg MAINTENANCE: SEVO 1 mac REM 0.1–0.5 mcg/kg/min	PCA: tramadol 500 MG basal infusion 3 mL/h bolus 3 mL lockout time 20 min	Postoperative Pain
Chen CK (2013) [43]	Hong Kong	21–60	I-II	ropivacaine 20 mL 3.75 mg/mL	no block	Preoperative	PREMEDICATION: oral MDZ 7,5 mg INDUCTION: FNT 2mcg/kg PRP 1–2 mg/kg Vecuronium 0.1 mg/kg MAINTENANCE: SEVO; vecuronium 0.05 mg/kg	if VAS score more than 4, morphine 0.05 mg/kg	Morphine Consumption
Tolchard S (2014) [44]	England	> 16	I-II	bupivacaine 1 mg/kg	bupivacaine 1 mg/ kg port site	Postoperative	INDUCTION: PRP 2.5 mg/kg FNT 3 mcg/kg atracurium 0.6 mg/kg	intraoperative: paracetamol 15–20 mg/kg diclofenac 0.5 mg/kg, postoperative: FNT 20 mcg boluses	Postoperative Pain
Breazu CM (2016) [45]	Romania	> 18	I-II	saline 20 mL	bupivacaine 0.25% 20 mL	Preoperative	PREMEDICATION: MDZ 7.5 mg po INDUCTION: PRP 2 mg/kg FNT 2 mcg/kg; ROC 0.6 mg/kg or atracurium 0.5 mg/kg MAINTENANCE: SEVO 1–2 MAC FNT 100 mcg when needed	Acetaminofen 15–20 mg/kg and 1 g iv at 8 h, pethidine 20–40 mg boluses and 20–40 mg iv every 3 hours	Postoperative Pain
Huang SH (2016) [46]	China		I-II	ropivacaine 15 mL 0.375%	no block	Preoperative	INDUCTION: midozolam 0.05 mg/kg PRP 2% REM ROC 0.6 mg/kg MAINTENANCE: PRP 2 mcg/mL REM 2–4 ng/mL ROC if needed	40 mg iv parecoxib, iv sulfentanil 5–10 mcg	Postoperative Pain
Saliminia A (2015) [47]	Iran			bupivacaine 0.5% 30 mL + 2 mL saline	32 mL saline 0.9%	Postoperative	INDUCTION: PRP 2 mg/kg, FNT 3 mcg/kg, atracurium 0.6 mg/kg MAINTENANCE: PRP 80–100 mcg/kg/min, FNT 1 mcg/kg/ 30 min, atracurium 0.3 mg/kg/30 min	PCA: FNT 50 mcg bolus; lock out time 8 min	Postoperative Opioid Consumption
Ali L (2018) [48]	Pakistan	20–60	I-II	0.5% bupivacaine (1 mg/kg),	0.5% bupivacaine (1 mg/kg),	Postoperative	INDUCTION:iv Nalbuphine 0.1 mg/kg, PRP 2–2.5 mg/kg iv MAINTENANCE isoflurane in 50% oxygen with air	Nalbuphine 2 mg iv	Postoperative Pain
Bava P (2016) [49]	India	18–65	I-II	0.375% ropivacaine	0.25% bupivacaine	Preoperative	INDUCTION:PRP 2 mg/kg + FNT 2 μg/kg + atracurium 0.5 mg/kg MAINTENANCE: isoflurane	Morphine 0.05 mg/kg boluses given until the VAS score < 4 + PCA (1 mg bolus) + as rescue analgesia if VAS > 3 0.1 mg/kg of morphine I	Postoperative Pain
Paudal B (2022) [50]	Nepal	18–60	I-II	bupivacaine 20 mL 0.25%	bupivacaine 20 mL 0.25%	Postoperative	PREMEDICATION: ranitidine 150 mg po INDUCTION: MDZ 0,02 mg/kg FNT 2 mcg/kg PRP 2.5 mg/kg vecuronium 0.1 mg/kg MAINTENANCE: isoflurane 1–1.5% intermittent doses of ROC		Postoperative Pain
Emile SH (2022) [51]	Egypt	N/A	I-II	10 ml bupivacaine (0.25%) + lidocaine (%2) + 10 ml saline	no block	Postoperative	INDUCTION: PRP 2 mg/kg, atracorium 1 mg/kg, FNT 1 μg/kg	IV 1000 mg Paracetamol, if VAS > 4 iv diclofenac for rescue analgesic	postoperative pain
Choi Y (2017) [52]	Korea	19–70	I-II	20 mL of ropivacaine 0.2%	no block	Preoperative	PREMEDICATION: glycopyrrolate (0.003 mg/kg) and MDZ (0.05 mg/kg) INDUCTION: thiopental sodium (5 mg/kg) and REM (1 μg/kg/min) ROC (0.8 mg/kg) MAINTENANCE: Desflurane (6 vol%) REM 0.5–1 μg/kg/min	IV-PCA: 100 mL of normal saline mixed with oxycodone (40 mg) and ketorolac (180 mg). The basal infusion rate was set to 1 mL/h, the bolus dose was 1 mL, and the lockout time was 15 min	postoperative pain
Prajapati K (2022) [53]	India	N/A	I-II	20 mL of 0.375% ropivacaine	no block	Postoperative	INDUCTION: FNT 2 mcg/kg, PRP 1.5–2.5 mg/kg and atracurium 0.5 mg/kg. MAINTENANCE: nitrous oxide (60%) and isoflurane (MAC: 0.8–1.2) in oxygen.	Paracetamol 1 g iv six hourly and inj. Diclofenac (75 mg/mL) 1 mL diluted in 100 mL normal saline iv 12 hourly. VAS ≥ 4 IV tramadol at an incremental dose of 2 mg/kg as rescue analgesia	postoperative pain
Ergin A (2021) [54]	Turkey	18–74	I-III	40 ml of bupivacaine 0.25%	no block	Preoperative	INDUCTION: 2–3 mg/kg PRP, 2 μg/kg FNT, and 0.6 mg/kg ROC MAINTENANCE:1.5–2% SEVO	VAS score of > 4 50 mg tramadol IV, and those with ongoing high pain after 30 min 100 mg tramadol, tenoxicam 20 mg IV postop 8 h, and 25 mg of meperidine IV as salvage analgesic	postoperative pain

Abbreviations: *MDZ* midazolam, *SUF* sufentanil, *REM* remifentanil, *ETM* etomidate, *ROC* rocuronium, *PRP* propofol, *PCA* patient-controlled analgesia, *FNT* fentanil, *SEVO* sevoflurane, *VAS* visual analog scale, *NRS* numerical rating scale

**Fig. 2:**
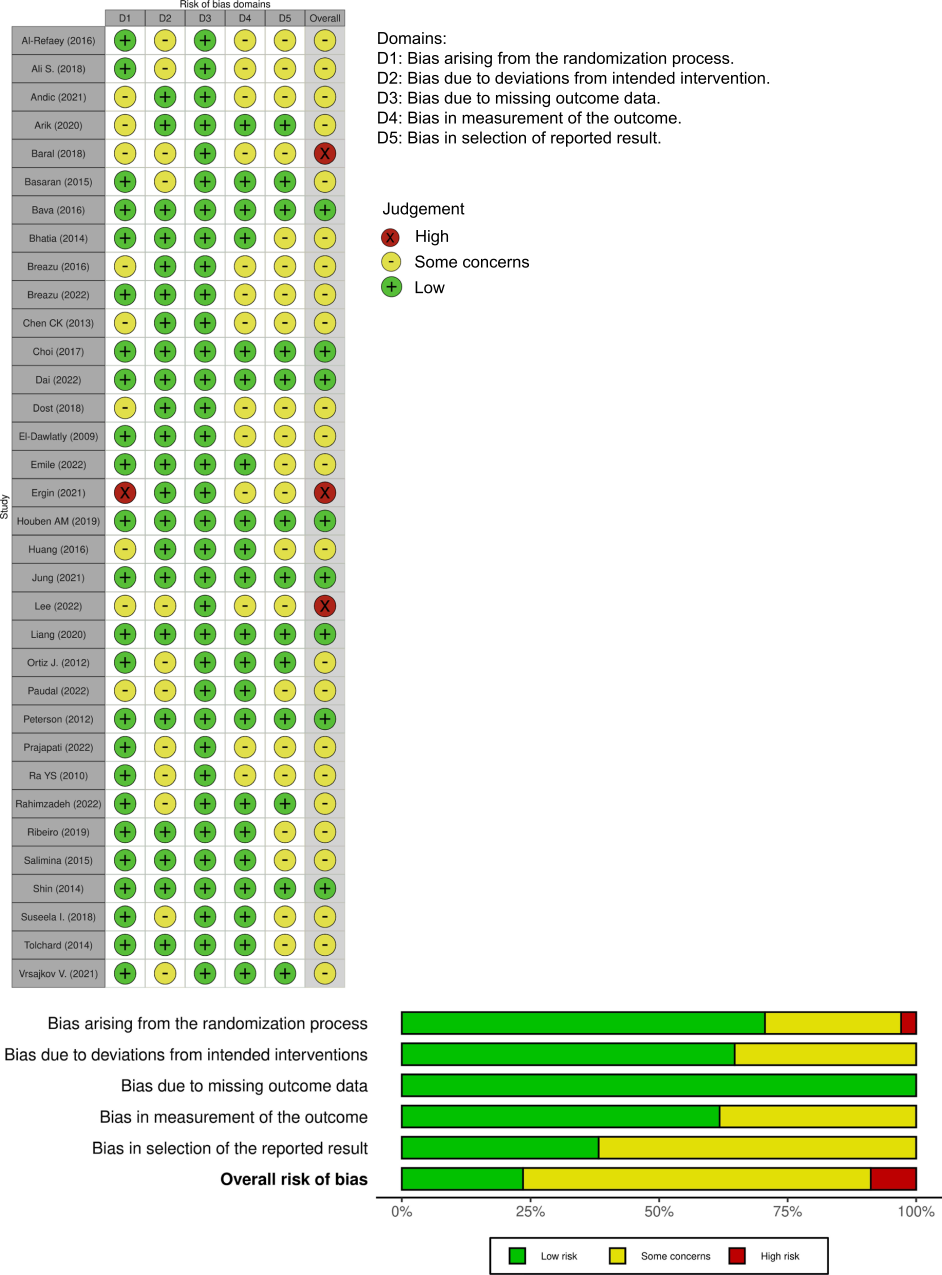
Bias assessment. An overview of the ROB2 (Risk of Bias 2) assessment is presented in this figure

In total, 22 of the included studies compared pre-operative TAP block with a control [22, 23, 25, 27–29, 31, 33, 35–43, 45, 46, 49, 52, 54], 11 evaluated the effect of postoperative TAP block compared with a control block [24, 26, 30, 32, 34, 44, 47, 48, 50, 51, 53], and only one directly compared pre-operative TAP block with postoperative TAP block [11]. A total of 2317 patients were enrolled in the included trials: 456 patients were randomised to the postoperative TAP block group, 773 to the pre-operative TAP block group, and the remaining to the control group. According to the risk of bias assessment, eight studies had a low risk of bias and three had a high risk. The 23 studies raised some concerns (Fig. 2). The criteria we used to assign the risk of bias judgments can be found in Supplementary Document 3.

### Outcomes

Most evidence arises from indirect comparisons, given that only one trial directly compared pre-operative and postoperative TAP blocks. While the cumulative effects of direct and indirect evidence are shown in the following section, the contribution of direct evidence for each comparison is shown in in Supplementary Document 4.

### Publication Bias

There was evidence of publication bias only for PONV outcomes (Egger’s test, *p* = 0.015). All funnel plots are shown in Supplementary Document 2.

### Main outcome: postoperative opioid consumption at 24 hours

**Fig. 3:**
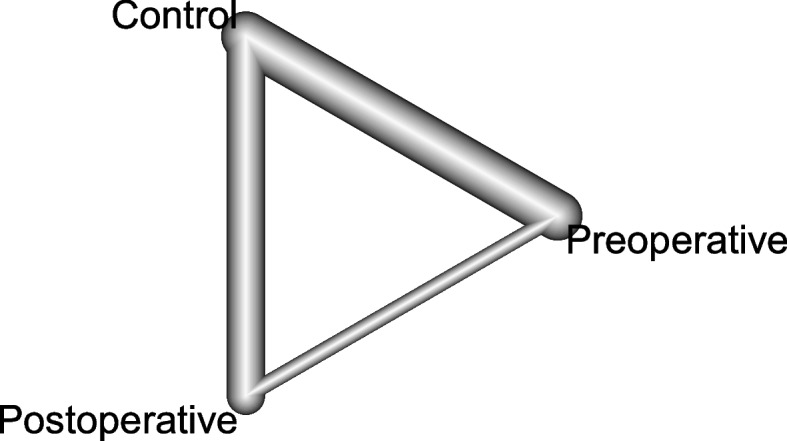
Network plot for intravenous morphine equivalents (mg) in the first 24 h. Each technique is represented at each corner of the polygon. The widths of the lines connecting interventions are proportionate to the number of trials assessing the comparisons

Postoperative opioid consumption was evaluated in 25 studies. A graphical representation of the network is shown in Fig. 3. Both postoperative and pre-operative TAP blocks resulted in a better reduction in opioid consumption at 24 h than the control (Table 2). However, the postoperative TAP block was superior to the pre-operative TAP block (MD 2.02, 95% CI 0.87. to 3.18, I^2^ 98.6%, *p* < 0.001) (Table 2). Using the GRADE assessment, we rated the quality of evidence as low, given both the high statistical heterogeneity and the major contribution of indirect evidence to the results.

**Table 2:** Postoperative vs. preoperative comparison

	k		MD (95% CI)	*p*-value	I2	Tau2
MME 24 h	25	Preoperative	2.02 (0.87;3.18)	< 0.001	98.6%	1.124
		Control	3.86 (2.90;44,82)	< 0.001		
Pain (0–3)	28	Preoperative	0.17 (−0.49;0.83)	0.621	97.8%	0.661
		Control	1.70 (1.13;2.27)	< 0.001		
Pain 12 h	21	Preoperative	0.30 (−0.44; 1.03)	0.429	93.6%	0.697
		Control	1.43 (0.81;2.05)	< 0.001		
Pain 24 h	26	Preoperative	0.36 (−0.14;0.87)	0.157	96.3%	0.356
		Control	1.06 (0.62;1.50)	< 0.001		
	k		OR (95% CI)	*p*-value	I2	Tau2
PONV	21	Preoperative	2.21 (1.20;4.08)	0.011	37.8%	0.197
		Control	2.26 (1.36;3.76)	0.002		

Postoperative TAP block is the reference group. “k” refers to the number of studies included in the analysis

### Postoperative pain

**Fig. 4:**
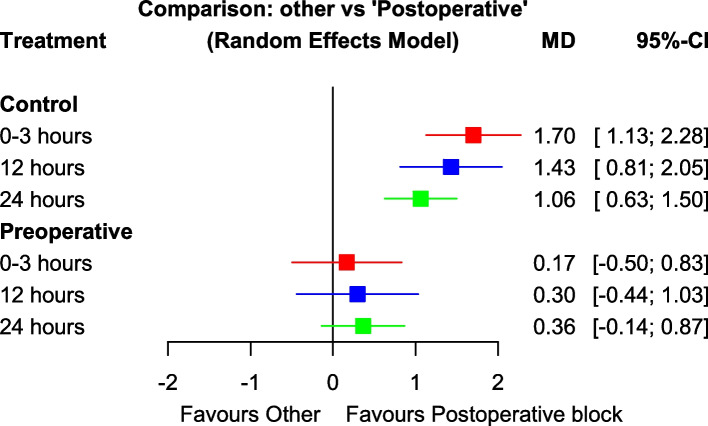
Forest plots for the pain scores. This figure displays the forest plots for the pain scores, providing a graphical representation of the results from individual studies

Pain in the first 3 hours was evaluated in 28 studies. Meanwhile, it was reported in 21 and 26 studies at 12 and 24 postoperative hours, respectively. Both techniques were superior to the control in providing better pain control at all times (Table 2). However, neither pre-operative nor postoperative TAP blocks were superior to each other at any of the considered time points (Table 2). A comprehensive summary of the results for this outcome are depicted in Fig.4. The postoperative TAP block was always ranked as the best intervention using SUCRA (Table 3). Using the GRADE assessment, we rated the quality of evidence as low, given both the high statistical heterogeneity and the major contribution of indirect evidence to the results.

**Table 3:** SUCRA

	MME	Pain (0–3)	Pain 12	Pain 24	PONV
Preoperative	2 (0.500)	2 (0.655)	2 (0.607)	2 (0.539)	2 (0.275)
Postoperative	1 (0.999)	1 (0.845)	1 (0.892)	1 (0.960)	1 (0.996)
Control	3 (0.000)	3 (0.000)	3 (0.000)	3 (0.000)	3 (0.228)

*PONV* Postoperative nausea and vomiting

### PONV

PONV was evaluated in 21 studies. The highest reduction in PONV was obtained with the postoperative TAP block (Table 2). Using the GRADE assessment, we rated the quality of evidence as low, given the major contribution of indirect evidence to the results, moderate statistical heterogeneity (I^2^ = 37.8%), and possible publication bias.

## Discussion

This systematic review and meta-analysis evaluated the analgesic effects of pre-operative and postoperative TAP blocks in patients undergoing LC. Based on 34 randomised controlled trials, we found that postoperative TAP block reduced postoperative 24-hour opioid consumption and PONV compared to pre-operative TAP block. The VAS scores were similar in the two groups.

Pain following laparoscopy has somatic, visceral, and referral components. Somatic pain arises from skin incisions during surgical port insertion, stretching of the abdominal wall due to CO_2_ insufflation, and surgical stimulation of the parietal peritoneum. It travels across the thoracolumbar spinal nerves (T6-L1), leading to sharp and well-localised pain rather than dull pain. In contrast, tissue traction, compression, or surgical dissection of the abdominal organs causes diffuse and dull visceral pain [1, 55]. This latter noxious impulse is transmitted by autonomic nerves (T5–9, greater splanchnic nerve, celiac ganglion, and vagus) [56, 57]. Furthermore, pneumoperitoneum and gallbladder stimulation may cause diaphragm inflammation, activating the phrenic nerve (C3–5) and causing referred shoulder pain [55]. The most significant component, in this case, is somatic pain [1].

As a component of multimodal therapy, TAP block guarantees somatic analgesia in the anterolateral abdominal wall by blocking the anterior branches of the thoracolumbar spinal nerves at different levels (T6-L1) [17]. Two meta-analyses compared TAP block to local anaesthetic wound infiltration or the control group, proving superior 24-hour postoperative analgesia [6, 58]. Pre-emptive analgesia blocks the noxious stimuli before they arise. Therefore, it is thought to be more effective than postoperative analgesia in preventing central sensitisation and incisional and inflammatory damage [59]. Therefore, we hypothesised that a pre-operative TAP block would reduce morphine consumption by providing pre-emptive analgesia and allowing more time for LA to diffuse in the fascial plane. Our results contradict these findings. A possible explanation for this discrepancy in the results could be that we included four approaches of TAP blocks, leading to different dermatomal coverages (subcostal, T6–9; lateral, T10–12; posterior, T9–12; and oblique-subcostal approach, T6-L1) [17]. Different dermatomal distributions ‘according to different approach of TAP block such as subcostal, lateral, and posterior’ among the studies may have inadvertently introduced variability in the degree of analgesia provided to the included study participants. This variability in dermatomal coverage could have directly influenced post-operative pain perception and, consequently, morphine consumption.

The optimum timing for performing a TAP block is still debated in the literature. A previous meta-analysis compared the analgesic effects of pre-operative and postoperative TAP blocks on postoperative pain in patients undergoing laparoscopic surgery [60]. The analysis found that pre-operative blocks reduced postoperative pain scores. In contrast, a retrospective study showed that 20 mL of 0.25% plain bupivacaine used for postoperative and pre-operative TAP block had a slightly similar effectiveness in reducing intravenous opioid consumption in the postoperative period. In patients that underwent postoperative TAP block, there was a decreased use of patient controlled analgesia (PCA) but a higher amount of morphine consumption. There was no difference between the groups regarding the duration of PCA or intravenous and oral opioid use [61]. In the same study, Kalu et al. proved better long-term outcomes (opioid prescribed at the discharge and amount of opioid) in the postoperative TAP group.

This network meta-analysis suggests that performing the block in the postoperative period may reduce 24-hour postoperative opioid consumption and PONV and may be slightly superior in terms of postoperative pain scores. Most of the evidence collected in this meta-analysis was indirect. However, a randomised controlled study comparing the timing of the block on postoperative outcomes found that performing a TAP block in the postoperative period significantly decreased postoperative opioid consumption, PONV, and pain scores at rest and during coughing [11]. Multimodal analgesia often relies heavily on opioids, which carry the risk of adverse effects, including nausea, constipation, respiratory depression, and the potential for addiction. In contrast, opioid-sparing analgesia techniques, such as the utilization of TAP blocks, aim to minimize opioid consumption while effectively controlling pain, reducing opioid-related side effects, and expediting postoperative recovery. By reducing opioid usage, patients experience improved pain control, faster return of bowel function, decreased length of hospital stay, and a quicker return to their normal daily activities [8].

Although there is still no agreement to define a clinically significant power of intervention [62], the overall reduction in opioid consumption found in this network meta-analysis was modest (− 2.23 mg), suggesting a small effect. However, considering the concomitant reduction in opioid-related PONV and postoperative pain scores, performing postoperative TAP block may be beneficial. Further studies are needed to confirm our findings, given the relatively low quality of evidence.

In addition, a TAP block requires a high-volume injection in a relatively highly vascularised area. After administration, peak plasma concentrations of ropivacaine and lidocaine were reached at the 30th and 15th minutes, respectively [63, 64]. Considering that the most intense pain occurs in the early postoperative hours, it may be more reasonable to perform a TAP block postoperatively.

Recent clinical findings suggest that the duration of a single shot TAP block can exceed 12 hours, with benefits reported up to 24–48 hours postoperatively for the posterior approach [65]. However, Støving et al. [66] reported in healthy volunteers a high variability effect of the block in terms of cutaneous sensory block area and block duration, which did not exceed 10 hours. In a pharmacokinetic study conducted by Trabelsi et al. [67] the mean elimination half-life of bupivacaine was 8.75 hours after the block. This result suggests a potentially decremental effect over 24 hours related to the metabolism and clearance of the local anaesthetic. The fact that the postoperative TAP block was closer to the assessment of outcomes and the peak of postoperative pain may partially explain its greater effectiveness.

Our meta-analysis is subject to several limitations; thus, caution should be exercised when interpreting our results. Firstly, there was considerable variation in the concentration, dose, and type of local anaesthetic used across the different TAP blocks included in the studies. Secondly, we encompassed all types of TAP blocks, which could introduce heterogeneity and impact the overall findings. Thirdly, the majority of the reported evidence is based on indirect comparisons, as only one study provided a direct comparison. Fourthly, it is important to acknowledge that data pertaining to block performance time and block dermatomal assessment were not available in the included studies. The absence of perioperative data in many of the studies may have influenced the quality of our analysis [68, 69]. Lastly, we observed significant heterogeneity in our analysis, which may affect the reliability and generalizability of the results.

## Conclusions

This systematic review and meta-analysis demonstrates that both pre-operative and postoperative TAP blocks are effective in reducing postoperative opioid consumption and pain scores. The postoperative TAP block appears to have a slight superiority and effectiveness in reducing 24-hour postoperative opioid consumption and PONV. However, it is essential to note that a high level of heterogeneity in the results may limit the robustness of our findings. Therefore, future studies on this topic will be of paramount importance to further validate and strengthen these results.

### Supplementary Information

**Additional file 1: Supplementary material 1.** Search Strategy.

**Additional file 2: Supplementary material 2.** Funnel Plots.

**Additional file 3: Supplementary Document 3.** Risk of bias assessment.

**Additional file 4: Supp. 4.** Proportion of direct evidence for each comparison.

## Data Availability

The datasets used and/or analysed during the current study available from the corresponding author on reasonable request.

## Abbreviations

TAP: Transversus Abdominis Plane
LC: Laparoscopic cholecystectomy
RCT: Randomized Controlled Trial
PONV: Postoperative Nausea And Vomiting
SUCRA: Surface Under The Cumulative Ranking Curve
PRISMA: Preferred Reporting Items for Systematic Reviews and Meta-Analyses
PICOS: Population, Intervention, Comparison, Outcomes and Study
PCA: Patient Controlled Analgesia
