# Supplementary material for: Analgesic benefits of pre-operative versus postoperative transversus abdominis plane block for laparoscopic cholecystectomy: a frequentist network meta-analysis of randomized controlled trials
Source: BMC Anesthesiol. 2023 Dec 12;23:408. doi: 10.1186/s12871-023-02369-6 (PMC10714465; doi:10.1186/s12871-023-02369-6)
Supplement: Supplementary file 1 — Additional file 1: Supplementary material 1. Search Strategy. [file 12871_2023_2369_MOESM1_ESM.docx]

Supplementary material 1. Search Strategy

| Pubmed |
| --- |
| (cholecystectomy AND (lps OR laparoscopic)) AND ((TAPB OR transversus abdominis block OR transverse abdominis block) OR (infiltration OR wound infiltration OR Trocar site infiltration))(randomizedcontrolledtrial[Filter]) |
| CENTRAL and Embase |
| (cholecystectomy AND (lps OR laparoscopic)) AND ((TAPB OR  transversus abdominis block OR transverse abdominis block) OR (infiltration OR  wound infiltration OR Trocar site infiltration)) in Title Abstract Keyword - (Word  variations have been searched) |
| Scopus |
| ( cholecystectomy AND laparoscopy ) AND ( tabp OR transversus AND abdominis AND plane AND block OR transvers AND abdominis AND plane AND block OR transversis AND abdominis AND plane AND block ) AND ( wound AND infiltration OR infiltration OR trochar AND infiltration ) SUBJAREA ( medi ) AND ( LIMIT-TO ( DOCTYPE , "ar" ) ) |
| Web of Science |
| (ALL=( laparoscopic cholecystectomy) OR ALL=(lps cholecystectomy)) AND  ((ALL=(transversus abdominis plane block) OR ALL=(transversis abdominis plane block) OR ALL=(TAPB) OR ALL=(infiltration) OR ALL=(wound infiltration) OR ALL=(trocar site infiltration)) |
