# Supplementary material for: Analgesic benefits of pre-operative versus postoperative transversus abdominis plane block for laparoscopic cholecystectomy: a frequentist network meta-analysis of randomized controlled trials
Source: BMC Anesthesiol. 2023 Dec 12;23:408. doi: 10.1186/s12871-023-02369-6 (PMC10714465; doi:10.1186/s12871-023-02369-6)
Supplement: Supplementary file 2 — Additional file 2: Supplementary material 2. Funnel Plots. [file 12871_2023_2369_MOESM2_ESM.pdf]

**Supplementary material 2. Funnel Plots**

**Main Outcome- Postoperative opioid consumption at 24 hours**

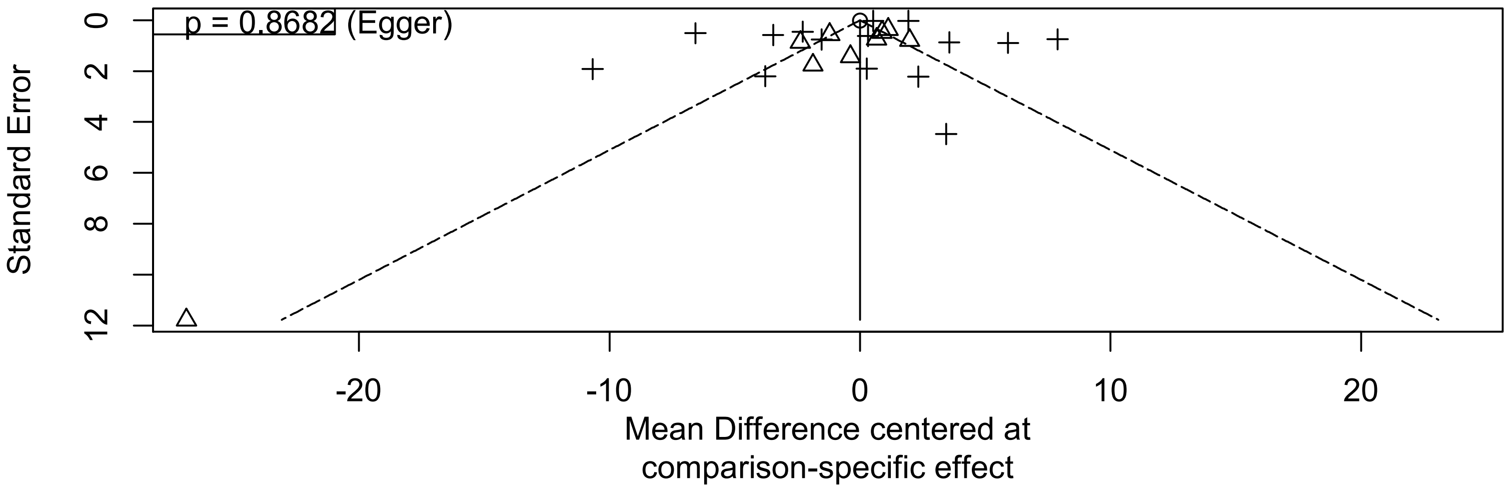

**Postoperative pain at 0-3 hours**

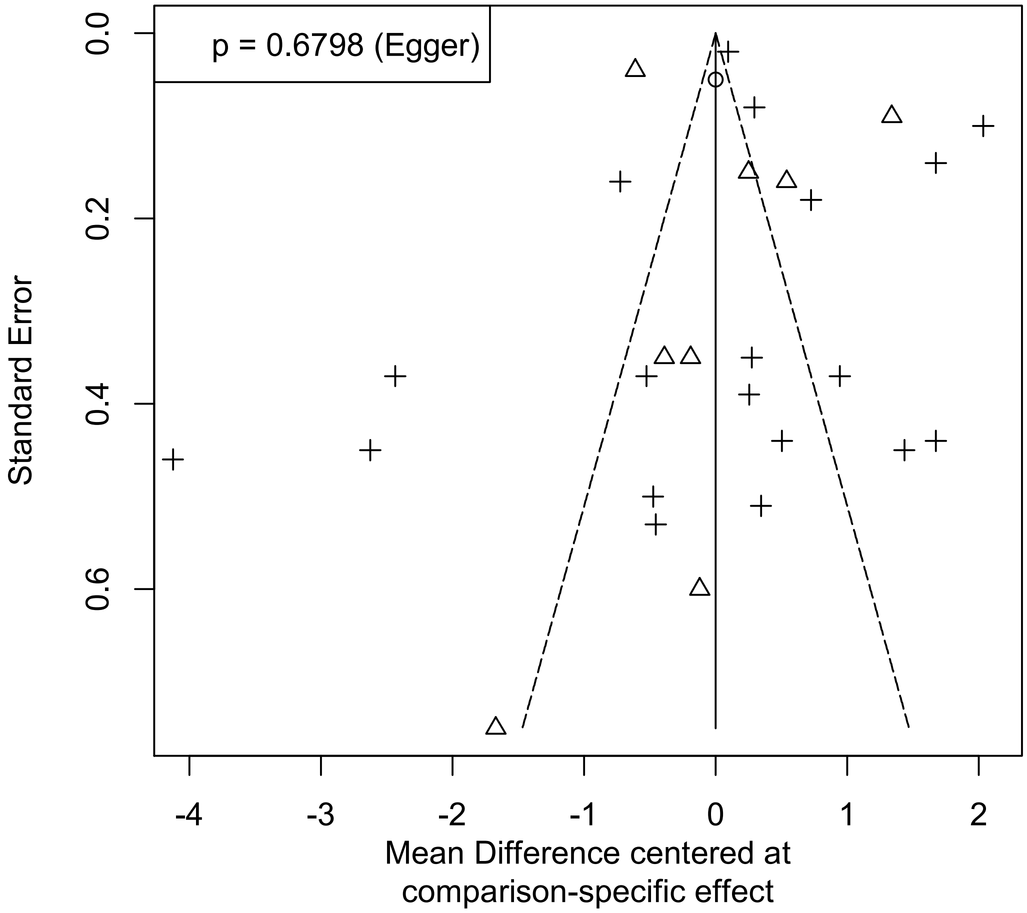

**Postoperative pain at 12th h**

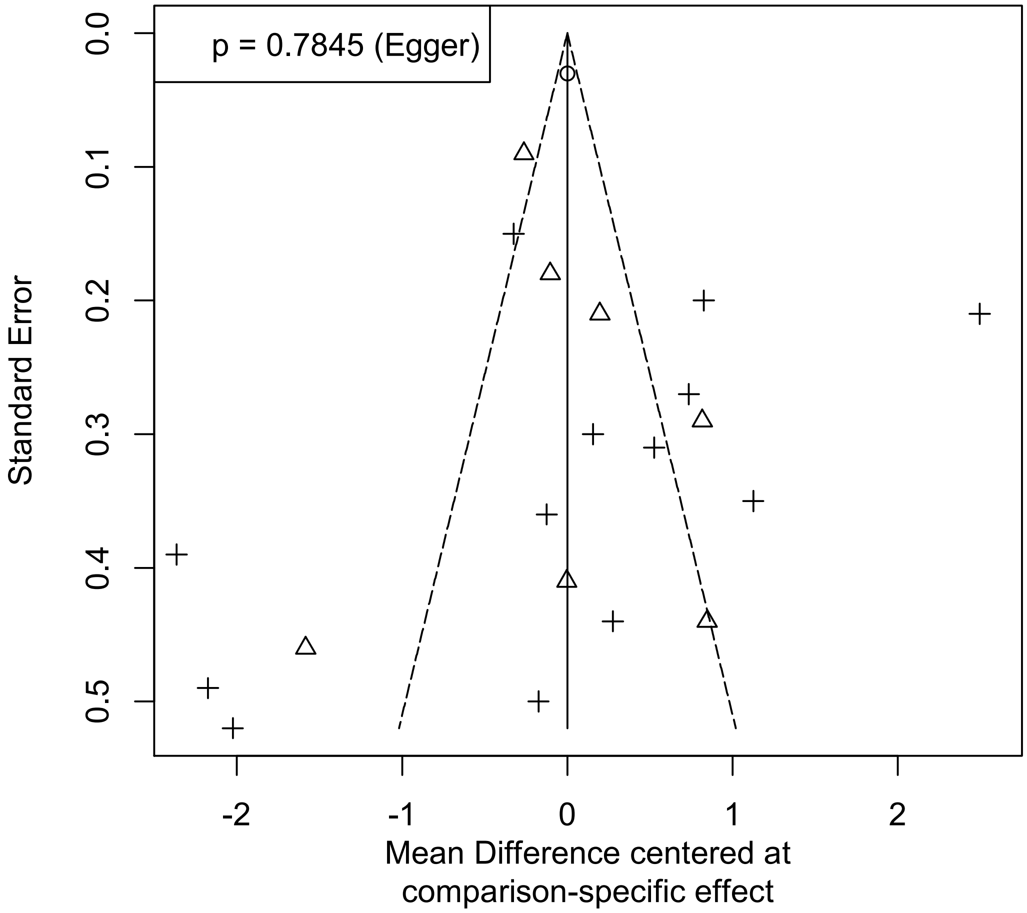

**Postoperative pain at 24th h**

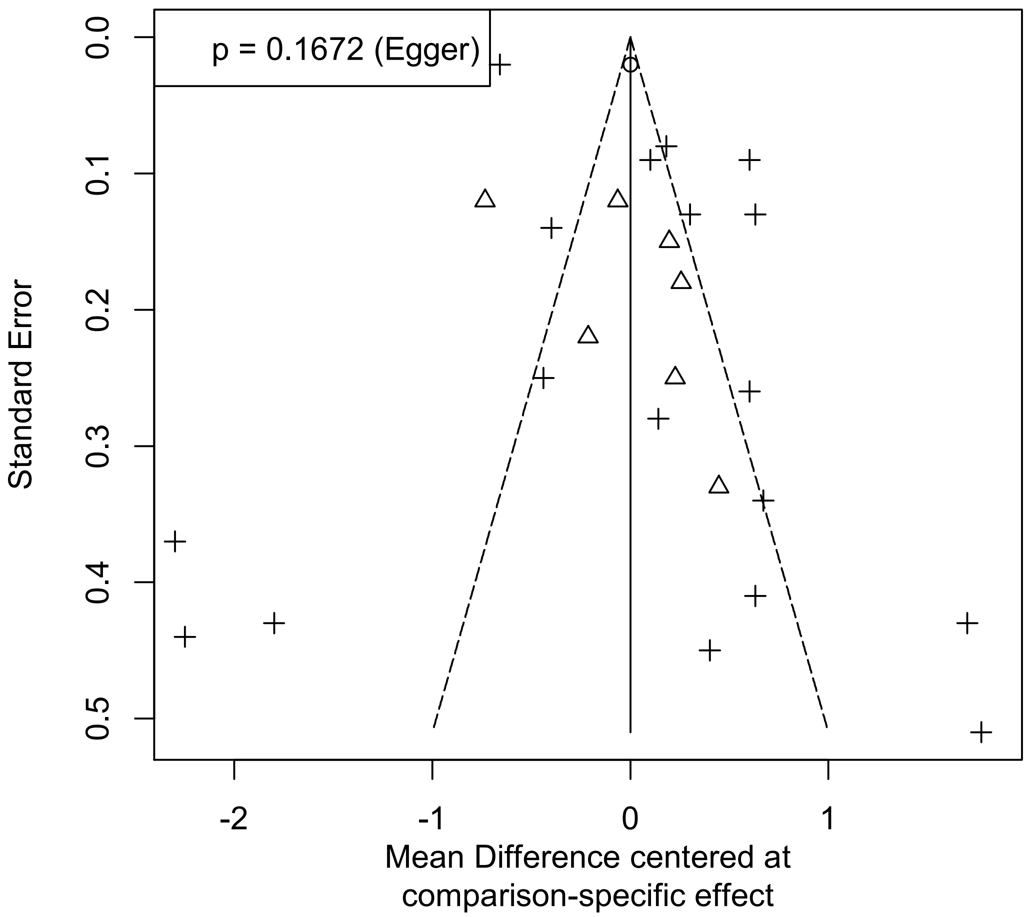

**PONV**

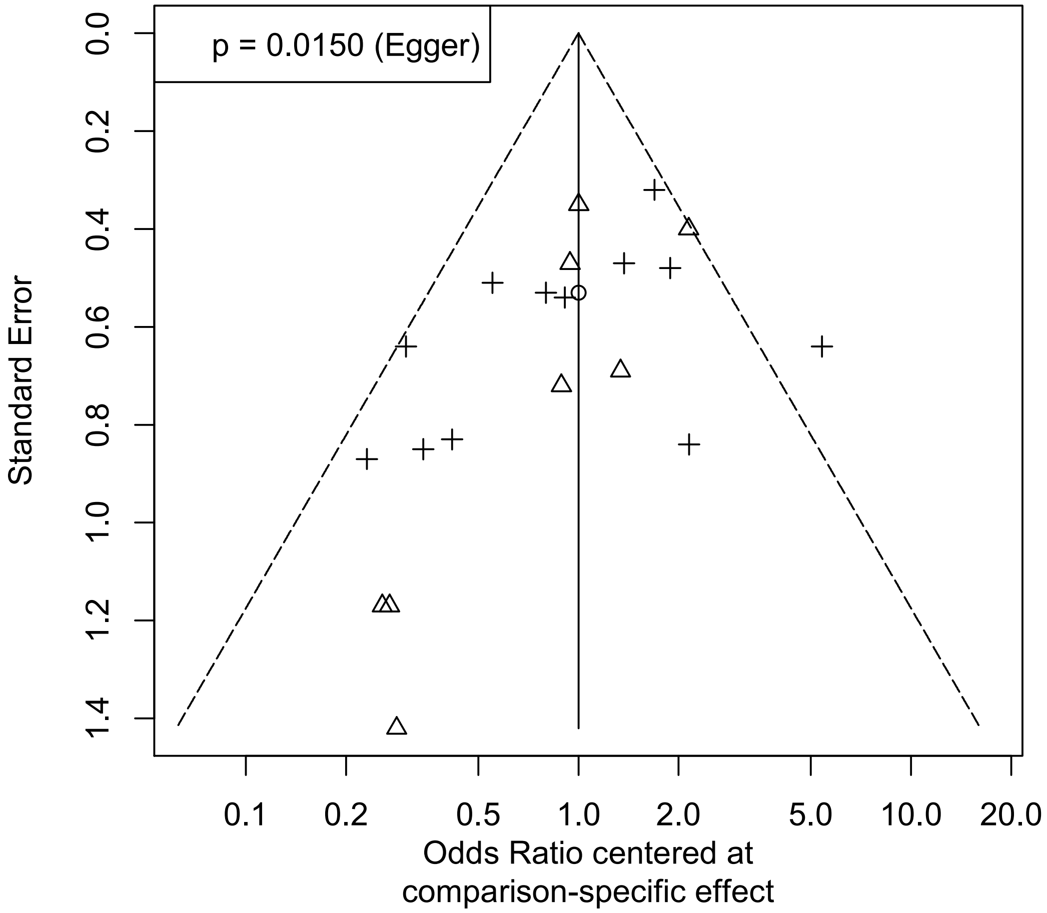
p = 0.0150 (Egger)
Standard Error
0.0
0.2
0.4
0.6
0.8
1.0
1.2
1.4
0.1
0.2
0.5
1.0
2.0
5.0
10.0
20.0
Odds Ratio centered at
comparison-specific effect
