# Supplementary material for: Analgesic benefits of pre-operative versus postoperative transversus abdominis plane block for laparoscopic cholecystectomy: a frequentist network meta-analysis of randomized controlled trials
Source: BMC Anesthesiol. 2023 Dec 12;23:408. doi: 10.1186/s12871-023-02369-6 (PMC10714465; doi:10.1186/s12871-023-02369-6)
Supplement: Supplementary file 3 — Additional file 3: Supplementary Document 3. Risk of bias assessment. [file 12871_2023_2369_MOESM3_ESM.docx]

**Supplementary Document 3. Risk of bias assessment.**

Ergin (2021)

|  |  |
| --- | --- |
| D1 | Non adequate concealment. Not stated how randomization and allocation was performed. |
| D2 | Personnel and patients were not aware of intervention. Appropriate analysis to estimate the effect of assignment. |
| D3 | Outcome data for all participants, a little discrepancy due to the gender. |
| D4 | No information on whether the outcome assessor was blinded to the intervention |
| D5 | No information regarding trial analysis performed according to a prespecified plan |
| Overall | High risk |

Prajapati (2022)

|  |  |
| --- | --- |
| D1 | Adequate concealment. A random component was used in the sequence generation process. Envelopes containing drugs were used appropriately. No imbalances are apparent. |
| D2 | Although it is stated double blind, it is not clear how the blinding process is performed. |
| D3 | Outcome data for all participants |
| D4 | No information on whether the outcome assessor was blinded to the intervention |
| D5 | No information regarding trial analysis performed according to a prespecified plan |
| Overall | Some concerns |

Choi (2017)

|  |  |
| --- | --- |
| D1 | Adequate concealment. A random component was used in the sequence generation process. |
| D2 | Personnel and patients were not aware of intervention. Appropriate analysis to estimate the effect of assignment. |
| D3 | Outcome data for all participants |
| D4 | Outcome assessor blinded to the intervention |
| D5 | Trial analysis was performed according to a prespecified plan (https://cris.nih.go.kr/cris/search/detailSearch.do?seq=9952&search_page=L) |
| Overall | Low risk |

Emile (2022)

|  |  |
| --- | --- |
| D1 | Adequate concealment. A random component was used in the sequence generation process. |
| D2 | Personnel and patients were not aware of intervention. Appropriate analysis to estimate the effect of assignment. |
| D3 | Outcome data for all participants |
| D4 | Outcome assessor blinded to the intervention |
| D5 | Trial analysis was conducted following a pre-specified plan (NCT04276285); however, the authors modified the originally prespecified sample size from the first version (n=126) to the second version of the protocol (n=94). |
| Overall | Some concerns |

El-Dawlatly (2009)

|  |  |
| --- | --- |
| D1 | Adequate concealment. A random component was used in the sequence generation process. Envelopes containing drugs were used appropriately. No imbalances are apparent. |
| D2 | Personnel and patients were not aware of intervention. Appropriate analysis to estimate the effect of assignment. |
| D3 | Outcome data for all participants |
| D4 | No information on whether the outcome assessor was blinded to the intervention |
| D5 | No information regarding trial analysis performed according to a prespecified plan |
| Overall | Some concerns |

Ra YS (2010)

| D1 | Patients were randomly allocated into three groups |
| --- | --- |
| D2 | Patients were probably not aware of intervention. Personnel were probably aware because of the interventions. Deviation may have affected outcome. |
| D3 | 54/54 patients evaluated |
| D4 | No information if the outcome assessor is blinded to the intervention |
| D5 | Trial analysis was not performed according to a prespecified plan |
| Overall | Some Concerns |

Ortiz J. (2012)

|  |  |
| --- | --- |
| D1 | Patients were randomized with a randomization program. |
| D2 | Patients and nurses assessing pain scores were blinded with regard to group assignment. Members of the anesthesia and surgical team were not blinded. |
| D3 | Outcome data for almost all participants (n=80) |
| D4 | Pain scores were recorded by the PACU and floor nurses taking care of the patient without knowledge of patient group assignment |
| D5 | Trial analysis was performed according to a prespecified plan |
| Overall | Some concerns |

Peterson (2012)

|  |  |
| --- | --- |
| D1 | Adequate concealment. A random component was used in the sequence generation process. Envelopes containing drugs were used appropriately. No imbalances are apparent. |
| D2 | Personnel and patients were not aware of intervention. Appropriate analysis to estimate the effect of assignment. |
| D3 | Outcome data for all participants |
| D4 | Outcome assessor blinded to the intervention |
| D5 | Trial analysis was performed according to a prespecified plan (NCT01046071) |
| Overall | Low risk |

Chen CK (2013)

| D1 | No information on sequence concealment. No imbalances are apparent. |
| --- | --- |
| D2 | No deviations arose because of the trial context |
| D3 | Outcome data for all participants |
| D4 | No information on whether the outcome assessor was blinded to the intervention |
| D5 | No information regarding trial analysis performed according to a prespecified plan |
| Overall | Some concerns |

Bhatia (2014)

|  |  |
| --- | --- |
| D1 | Adequate concealment. A random component was used in the sequence generation process. Envelopes containing drugs were used appropriately. No imbalances are apparent. |
| D2 | Personnel and patients were not aware of intervention. Appropriate analysis to estimate the effect of assignment. |
| D3 | Outcome data for all participants |
| D4 | Outcome assessor blinded to the intervention |
| D5 | No information regarding trial analysis performed according to a prespecified plan |
| Overall | Some concerns |

Shin (2014)

| D1 | Adequate concealment. A random component was used in the sequence generation process. Envelopes containing drugs were used appropriately. No imbalances are apparent. |
| --- | --- |
| D2 | Personnel and patients were not aware of intervention. Appropriate analysis to estimate the effect of assignment. |
| D3 | Outcome data for all participants |
| D4 | Outcome assessor blinded to the intervention |
| D5 | Trial analysis was performed according to a prespecified plan (KCT0000404) |
| Overall | Low risk |

Tolchard (2014)

| D1 | A random component was used computer generated random numbers. No imbalances are apparent. |
| --- | --- |
| D2 | Patients and personnel were not aware of intervention. |
| D3 | Outcome for all participants. |
| D4 | Outcome assessor was blind to the intervention |
| D5 | Trial analysis was not performed according to a prespecified plan |
| Overall | Some concerns |

Basaran (2015)

|  |  |
| --- | --- |
| D1 | A random component was used. No imbalances are apparent. |
| D2 | Patients were not aware of intervention. Personnel were aware because of the trial context. Deviations couldn’t affect outcome and were balanced between the groups. |
| D3 | Outcome data for 76/81 participants. Probably the result was not biased by missing outcome data. |
| D4 | Outcome assessor blinded to the intervention |
| D5 | Trial analysis was performed according to a prespecified plan (protocol B.30.2.SEL.0.28.00.00/130) |
| Overall | Some concerns |

Salimina (2015)

| D1 | A random component was used computer generated random numbers. No imbalances are apparent. |
| --- | --- |
| D2 | Patients and personnel were not aware of intervention. |
| D3 | Outcome for all participants. |
| D4 | Outcome assessor was blind to the intervention |
| D5 | Trial analysis was not performed according to a prespecified plan |
| Overall | Some concerns |

Al-Refaey (2016)

| D1 | A random component was performed using closed envelope  technique. No imbalances are apparent. |
| --- | --- |
| D2 | Patients were probably not aware of intervention, personnel were aware because of the trial context. There was not an appropriate analysis. Deviations couldn’t affect outcome and were balanced between the groups. |
| D3 | Outcome data for all participants |
| D4 | Don’t know if the outcome assessor was blinded to the intervention. |
| D5 | Trial analysis was not performed according to a prespecified plan |
| Overall | Some concerns |

Bava (2016)

| D1 | Patients were randomly assigned to two groups using a computer generated table of random numbers which was enclosed in a sealed envelope and was opened by an anesthesiologist who was not involved  in the study. |
| --- | --- |
| D2 | Patients and personnel were not aware of intervention. |
| D3 | All participants were evaluated |
| D4 | Postoperative parameters were recorded either by anesthesiologist or by nursing staff who were blinded to the study groups. |
| D5 | The study protocol was approved by the Institutional Ethical Committee and registered at Clinical Trials Registry - India (CTRI) (CTRI/2014/09/004942, September 1, 2014). |
| Overall | Low risk of bias |

Breazu (2016)

| D1 | No information on sequence concealment. No imbalances are apparent. |
| --- | --- |
| D2 | Personnel and patients were not aware of intervention. Appropriate analysis to estimate the effect of assignment. |
| D3 | Outcome data for all participants |
| D4 | No informations if the outcome assessor is blinded to the intervention |
| D5 | Trial analysis was not performed according to a prespecified plan |
| Overall | Some concerns |

Huang (2016)

| D1 | No information on sequence concealment. No imbalances are apparent. |
| --- | --- |
| D2 | Patients were not aware of intervention. |
| D3 | Outcome for all participants. |
| D4 | Outcome assessor was blind to the intervention |
| D5 | Trial analysis was not performed according to a prespecified plan |
| Overall | Some concerns |

Ali S. (2018)

| D1 | A random component was used in the sequence generation process. No imbalances are apparent. |
| --- | --- |
| D2 | Patients were not aware of intervention but the personnel was. Appropriate analysis to estimate the effect of assignment. |
| D3 | Outcome data for all participants |
| D4 | No information on whether the outcome assessor was blinded to the intervention. |
| D5 | Trial analysis was not performed according to a prespecified plan |
| Overall | Some concerns |

Baral (2018)

| D1 | No information on sequence concealment. No imbalances are apparent. |
| --- | --- |
| D2 | Patients were probably not aware of intervention. Personnel were probably aware because of the interventions. Deviation may have affected outcome. |
| D3 | Outcome data for all participants |
| D4 | No informations if the outcome assessor is blinded to the intervention |
| D5 | Trial analysis was not performed according to a prespecified plan |
| Overall | High Risk |

Dost (2018)

|  |  |
| --- | --- |
| D1 | No information on sequence concealment. No imbalances are apparent. |
| D2 | No deviations arose because of the trial context |
| D3 | Outcome data for all participants |
| D4 | No information on whether the outcome assessor was blinded to the intervention |
| D5 | No information regarding trial analysis performed according to a prespecified plan |
| Overall | Some concerns |

Suseela I. (2018)

| D1 | A random component was used in the sequence generation process.No imbalances are apparent. |
| --- | --- |
| D2 | Patients were not aware of intervention; personnel were aware but were not involved in postoperative follow up of the patients. Appropriate analysis to estimate the effect of assignment. |
| D3 | Outcome data for all participants |
| D4 | Outcome assessor blinded to the intervention |
| D5 | Trial analysis was not performed according to a prespecified plan |
| Overall | Some concerns |

Houben AM (2019)

| D1 | Randomization was determined using a computer-generated list with a 1:1 allocation. |
| --- | --- |
| D2 | The study solutions were prepared by two investigators who were neither involved in the anesthetic care nor in data collection. Anesthesiologists responsible for the anesthetic management and data collection were unaware of group allocation. |
| D3 | Outcome data for all participants |
| D4 | Anesthesiologists responsible for the anesthetic management and  data collection were unaware of group allocation. |
| D5 | Trial analysis was performed according to a prespecified plan (NCT0339153) |
| Overall | Low risk of bias |

Ribeiro (2019)

|  |  |
| --- | --- |
| D1 | A random component was used in the sequence generation process. No imbalances are apparent. |
| D2 | Personnel and patients were not aware of intervention. Appropriate analysis to estimate the effect of assignment. |
| D3 | Outcome data for all participants |
| D4 | Outcome assessor blinded to the intervention |
| D5 | No information regarding trial analysis performed according to a prespecified plan |
| Overall | Some concerns |

Arik (2020)

|  |  |
| --- | --- |
| D1 | No information on sequence concealment. No imbalances are apparent. |
| D2 | Personnel and patients were not aware of intervention. Appropriate analysis to estimate the effect of assignment. |
| D3 | Outcome data for all participants |
| D4 | Outcome assessor blinded to the intervention |
| D5 | Trial analysis was performed according to a prespecified plan (NCT03543202) |
| Overall | Some concerns |

Liang (2020)

|  |  |
| --- | --- |
| D1 | Adequate concealment. A random component was used in the sequence generation process. Envelopes containing drugs were used appropriately. No imbalances are apparent. |
| D2 | Personnel and patients were not aware of intervention. Appropriate analysis to estimate the effect of assignment. |
| D3 | Outcome data for all participants |
| D4 | Outcome assessor blinded to the intervention |
| D5 | Trial analysis was performed according to a prespecified plan (ChiCTR-  TRC-14004193) |
| Overall | Low risk |

Andic (2021)

| D1 | No information on sequence concealment. No imbalances are apparent. |
| --- | --- |
| D2 | No deviations arose because of the trial context |
| D3 | Outcome data for all participants |
| D4 | No information on whether the outcome assessor was blinded to the intervention |
| D5 | No information regarding trial analysis performed according to a prespecified plan |
| Overall | Some concerns |

Jung (2021)

| D1 | Adequate concealment. A random component was used in the sequence generation process. Envelopes containing drugs were used appropriately. No imbalances are apparent. |
| --- | --- |
| D2 | Personnel and patients were not aware of intervention. Appropriate analysis to estimate the effect of assignment. |
| D3 | Outcome data for all participants |
| D4 | Outcome assessor blinded to the intervention |
| D5 | Trial analysis was performed according to a prespecified plan (KCT0004835) |
| Overall | Low risk |

Vrsajkov V. (2021)

|  |  |
| --- | --- |
| D1 | A random component was used in the sequence generation process. No imbalances are apparent. |
| D2 | Patients were not aware of intervention. Appropriate analysis to estimate the effect of assignment. |
| D3 | Outcome data for all participants |
| D4 | Outcome assessor blinded to the intervention |
| D5 | Trial analysis was performed according to a prespecified plan |
| Overall | Some concerns |

Breazu (2022)

| D1 | Adequate concealment. A random component was used in the sequence generation process. Envelopes containing drugs were used appropriately. No imbalances are apparent. |
| --- | --- |
| D2 | Personnel and patients were not aware of intervention. Appropriate analysis to estimate the effect of assignment. |
| D3 | Outcome data for all participants |
| D4 | No informations if the outcome assessor is blinded to the intervention |
| D5 | Trial analysis was not performed according to a prespecified plan |
| Overall | Some concerns |

Dai (2022)

| D1 | Adequate concealment. A random component was used in the sequence generation process. Envelopes containing drugs were used appropriately. No imbalances are apparent. |
| --- | --- |
| D2 | Personnel and patients were not aware of intervention. Appropriate analysis to estimate the effect of assignment. |
| D3 | Outcome data for all participants |
| D4 | Outcome assessor blinded to the intervention |
| D5 | Trial analysis was performed according to a prespecified plan (ChiCTR2000034001) |
| Overall | Low risk |

Lee (2022)

| D1 | No information on sequence concealment. No imbalances are apparent. |
| --- | --- |
| D2 | Patients were probably not aware of intervention. Personnel were probably aware because of the interventions. Deviation may have affected outcome. |
| D3 | Outcome data for all participants |
| D4 | No informations if the outcome assessor is blinded to the intervention |
| D5 | Trial analysis was not performed according to a prespecified plan |
| Overall | High Risk |

Paudal (2022)

| D1 | No information on sequence concealment. No imbalances are apparent. |
| --- | --- |
| D2 | No information on blinding. Appropriate analysis to estimate the effect of assignment. |
| D3 | Outcome data for all participants |
| D4 | Outcome assessor was blinded |
| D5 | Trial analysis was not performed according to a prespecified plan |
| Overall | Some concerns |

Rahimzadeh (2022)

| D1 | Patients were randomly assigned to two groups using a computer generated table of random numbers which was enclosed in a sealed envelope and was opened by an anesthesiologist who was not involved in the study. |
| --- | --- |
| D2 | Patients were not blinded. Appropriate analysis to estimate the effect of assignment. |
| D3 | Outcome data for all participants |
| D4 | Outcome assessor was blinded |
| D5 | The study protocol was approved by the Institutional Ethical Committee and registered IRCT20120814010599N26 |
| Overall | Some concerns |
