# Supplementary material for: Analgesic benefits of pre-operative versus postoperative transversus abdominis plane block for laparoscopic cholecystectomy: a frequentist network meta-analysis of randomized controlled trials
Source: BMC Anesthesiol. 2023 Dec 12;23:408. doi: 10.1186/s12871-023-02369-6 (PMC10714465; doi:10.1186/s12871-023-02369-6)
Supplement: Supplementary file 4 — Additional file 4: Supp. 4. Proportion of direct evidence for each comparison. [file 12871_2023_2369_MOESM4_ESM.docx]

Supp.4. Proportion of direct evidence for each comparison

|  | **MME 24h** | | **Pain (0-3h)** | | **Pain (12h)** | | **Pain (24h)** | | **PONV** | |
| --- | --- | --- | --- | --- | --- | --- | --- | --- | --- | --- |
|  | Postop | Control | Postop | Control | Postop | Control | Postop | Control | Postop | Control |
| **Preoperative** | 22% (1) | 92% (15) | 17% (1) | 95% (19) | 20% (1) | 93% (13) | 19% (1) | 94% (18) | 20% (1) | 92% (12) |
| **Postoperative** |  | 86% (9) |  | 88% (8) |  | 87% (7) |  | 87% (7) |  | 87% (8) |

NOTE: Percentage of direct evidence (number of studies). Postop: postoperative, PONV:Postoperative nausea and vomiting
